# Structural Defects Lead to Dynamic Entrapment in Cardiac Electrophysiology

**DOI:** 10.1371/journal.pone.0119535

**Published:** 2015-03-10

**Authors:** Oliver R. J. Bates, Bela Suki, Peter S. Spector, Jason H. T. Bates

**Affiliations:** 1 Boston University College of Engineering, 44 Cummington Mall, Boston, Massachusetts, 02215, United States of America; 2 University of Vermont College of Medicine, 89 Beaumont Avenue, Burlington, Vermont, 05405, United States of America; Gent University, BELGIUM

## Abstract

Biological networks are typically comprised of many parts whose interactions are governed by nonlinear dynamics. This potentially imbues them with the ability to support multiple attractors, and therefore to exhibit correspondingly distinct patterns of behavior. In particular, multiple attractors have been demonstrated for the electrical activity of the diseased heart in situations where cardioversion is able to convert a reentrant arrhythmia to a stable normal rhythm. Healthy hearts, however, are typically resilient to abnormal rhythms. This raises the question as to how a healthy cardiac cell network must be altered so that it can support multiple distinct behaviors. Here we demonstrate how anatomic defects can give rise to multi-stability in the heart as a function of the electrophysiological properties of the cardiac tissue and the timing of activation of ectopic foci. This leads to a form of hysteretic behavior, which we call dynamic entrapment, whereby the heart can become trapped in aberrant attractor as a result of a transient change in tissue properties. We show that this can lead to a highly inconsistent relationship between clinical symptoms and underlying pathophysiology, which raises the possibility that dynamic entrapment may underlie other forms of chronic idiopathic illness.

## Introduction

Biological function arises from the activities of networks of components, often cells and/or molecules that typically interact in highly dynamic and nonlinear ways. This may have important implications for human health because of the well-known ability of (even simple 2D) nonlinear dynamic systems to support multiple attractors [1], allowing them to potentially manifest a number of very different but stable behavior patterns. If a critical network involved in normal biological function can support more than one stable attractor, it is conceivable that one of these attractors could correspond to health and the others to various forms of pathology [2–5]. This raises the possibility that some chronic diseases might correspond to a structurally normal system operating in an abnormal manner, perhaps as a result of an inopportune interaction with the environment that pushed the system into the clutches of the pathological attractor at some point in the past. We refer to this situation as dynamic entrapment [3].

On the other hand, natural selection may have taken care to avoid dynamic entrapment in those networks critical to life by evolving them to have only a single stable attractor that corresponds to normal healthy function. If this is the case then such networks should exhibit evidence of design constraints toward this end. For example, in a previous study [5] we showed that Hopfield-like networks exhibit only a single attractor when their connectivity is sufficiently sparse. Sparse connectivity could thus be a design constraint used by Nature in order to prevent its critical biological networks from falling into the clutches of aberrant stable states. If this is generally true then the only way that disease can occur as a result of dynamic entrapment is if the network itself becomes damaged or abnormal.

Whether disease can manifest in structurally healthy biological systems via dynamic entrapment has some intriguing implications for diagnosis and therapy, but it still remains an open question waiting for the discovery of its first exemplar. There seems to be little doubt, however, that dynamic entrapment can exist in pathological states; an excellent example is provided by the demonstrated efficacy of cardioversion for treating cardiac arrhythmias [2]. That is, when the pattern of cardiac excitation is stable both in its aberrant form and immediately following cardioversion to sinus rhythm, the heart obviously must be able to support two stable dynamic states. On the other hand, sinus rhythm in the normal heart is very robust; while it is possible to transiently induce arrhythmias in a perfectly healthy heart by burst pacing, the return to sinus rhythm is rapid and enduring. The network of excitable cells comprising the healthy normal heart would thus seem to be constructed in such a way as to exhibit only one stable dynamic attractor, implying that arrhythmic disease via dynamic entrapment can only occur in a heart cell network that is somehow damaged either anatomically or physiologically.

This raises the question as to what network abnormalities in the heart might give rise to the potential for dynamic entrapment. Numerous computational model studies have demonstrated bifurcation behavior leading to reentrant patterns of electrical activity, in both homogeneous and heterogeneous media, and have generated aberrant activity using the equivalent of ectopic foci (i.e. well timed extraneous depolarization) [6–12]. While this is certainly a mechanism by which cardiac arrhythmias can arise clinically, it is not the only possibility. In particular, abnormalities in the conduction properties of the cardiac tissue represent a very important alternative pathway to arrhythmogenesis. Understanding how such abnormalities lead to arrhythmias that exemplify dynamic entrapment would serve as a basis on which to understand not only the arrhythmias themselves but also how the phenomenon of dynamic entrapment might manifest in other biological systems. Accordingly, in the present study we investigated how spatial heterogeneities in cardiac conduction can lead to dynamic entrapment, and how this might account for the sometimes confusingly episodic nature of certain cardiac arrhythmias.

## Methods

We used a previously described diffusion model for propagation in excitable media [13] consisting of electrically excitable cells interconnected by ohmic resistors through which the cells exchange charge (http://circep.ahajournals.org/content/suppl/2011/06/08/CIRCEP.110.961524.DC1.html). The difference equation governing the evolution of model cell *i*'s potential is given by Equation 1 [13]. Each model cell (representing a large group of myocytes) generates an action potential (AP) once it has accumulated sufficient charge from its neighbors. The AP profile for a given cell is determined by baseline upstroke velocity and repolarization slope, as well as by electrotonic currents resulting from non-uniform potential distributions in the tissue.

$$V_{i}\left[t+1\right]=\underset{neighborsj}{\sum }\frac{V_{j}\left[t\right]-V_{i}\left[t\right]}{{\text{C}}_{i}{\text{R}}_{ij}}+\Delta V_{R,i}\left[t\right]$$ Eq. 1

where

$$\Delta V_{R,i}\left[t\right]=\left\{\begin{array}{cc} \begin{array}{c} \begin{array}{c} sgn\left(V_{rest,i}-V_{i}\left[t\right]\right)\times \Delta V_{leak,i}\\ \Delta V_{up,i} \end{array}\\ \begin{array}{c} 0\\ \Delta V_{repol,i} \end{array} \end{array} & \begin{array}{c} \begin{array}{c} ifcelliisatrest\\ ifcelliisinupstrokephase \end{array}\\ \begin{array}{c} ifcelliisinplateauphase\\ ifcelliisinrepolarizationphase \end{array} \end{array} \end{array}\right.$$

In the present study, we considered 2-dimensional model tissues that consisted of some regions that conducted electrical excitation and other regions that did not. In the conducting regions all model parameters were spatially homogeneous, but we allowed the intercellular resistance, *R*, and the baseline repolarization slope, *ΔV*_*repol*_, to vary with time. *R* affects primarily the conduction velocity (*CV*) of waves of excitation in the model because it determines how rapidly an excitable cell can deliver charge to an unexcited neighbor. Increasing *R* makes it more difficult for a given cell to excite its neighbors, and conduction fails completely at a critical threshold. *ΔV*_*repol*_ affects primarily the action potential duration (*APD*). Increasing *ΔV*_*repol*_ (i.e. shortening *APD*) causes a cell to spend less time with an elevated potential relative to its unexcited neighbors, which decreases its capacity to bring these neighbors up to their excitation thresholds, and vice-versa. *R* and *ΔV*_*repol*_ together determine the wavelength (*WL* = *CV*.*APD*) of excitation. Reducing *WL* increases the likelihood that the tissue will sustain continuous patterns of circulating excitation known as reentry.

**Fig 1:**
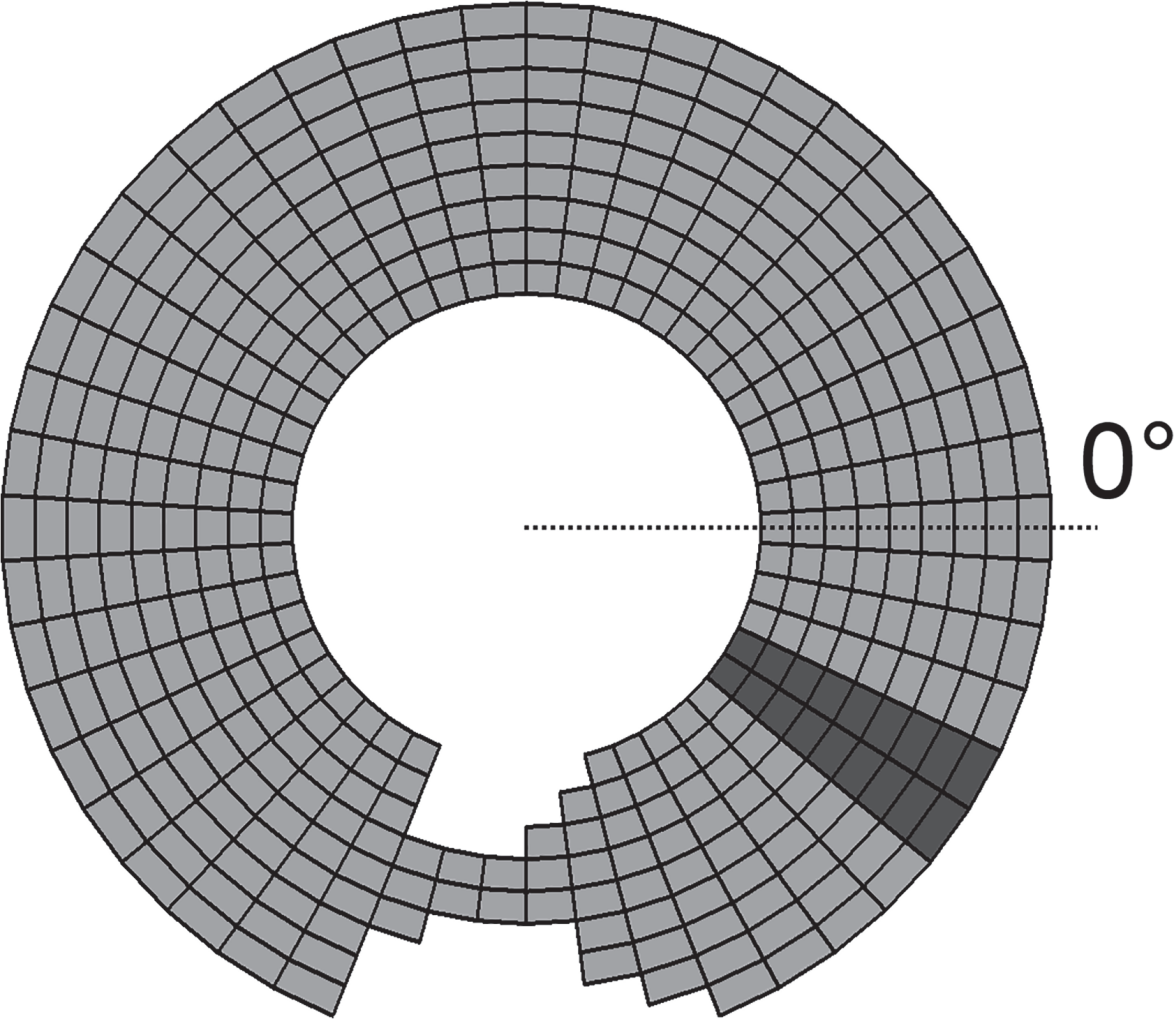
Model setup for simulating anatomically defined reentry. The tissue has a circumference of 50mm and a width of 9mm, and contains a pacemaker site (dark grey cells) and a narrowing “wedge” which allows for the possibility of unidirectional block due to asymmetric source-sink relationships.

It is well known that reentrant propagation must involve circuits with path lengths exceeding *WL* so that fronts of activation are not extinguished by impinging on their own refractory tails [14]. In the present study we achieve this with a two-dimensional annulus of model tissue having a path length longer than *WL*. This annulus includes an asymmetric narrowing of the tissue (located at −90 degrees in Fig. 1) such as might be created in an actual heart by a concealed accessory pathway [15]. At the site of tissue narrowing shown in Fig. 1, a wave front traveling clockwise round the circuit is progressively reduced from a full-width front down to only two excited cells. After traversing the isthmus, this narrow excitation front is quickly exposed to a full-width row of unexcited cells. If the two excited cells cannot generate enough current between them to excite the entire unexcited row then propagation will fail [16]. When the wave front travels in the reverse direction, however, the increase in the width of excitable tissue, and thus the need for additional current, is not as sudden and so propagation is more likely to be successful, giving rise to uni-directional block.

Activation is initiated by a band of spontaneously depolarizing pacemaker cells (located at −30 degrees in Fig. 1).

## Results

**Fig 2:**
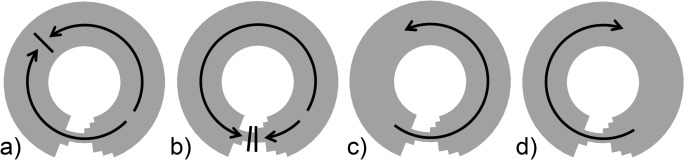
Stable system behaviors. (a) Sinus Rhythm with Clockwise Conduction, SR_cond_, requires that the wedge conducts in the clockwise direction; (b) Sinus Rhythm with Clockwise Block, SR_block_, requires that the wedge be non-conducting; (c) Counter-Clockwise Reentry, CCWR, requires that the wedge conducts in the counter-clockwise direction; (d) Clockwise Reentry, CWR, requires that the wedge conducts in the clockwise direction.

The model system shown in Fig. 1 gives rise to one of four possible stable behaviors, illustrated in Fig. 2. The first behavior, which we label *Sinus Rhythm with Clockwise Conduction* (SR_cond_), corresponds to the system being driven by the spontaneous depolarization of the pacemaker cells (Fig. 2A). Each depolarization gives rise to two wave fronts originating in the pacemaker cells and traveling in opposite directions around the circuit—one in the counter-clockwise direction (over the top of the circuit) and the other in the clockwise direction (through the wedge located on the bottom). These waves collide opposite the pacemaker site and annihilate, after which the system returns to rest and waits to be re-excited by the pacemaker cells. In analogy to a real heart, this sequence of electrical activity corresponds to one normal beat. The second behavior, which we label *Sinus Rhythm with Bi-directional Block* (SR_block_), is similar to SR_cond_ in that ongoing activity is still driven by the pacemaker cells (Fig. 2B). However, in this rhythm, the wedge is non-conducting in both directions so that the clockwise wave is blocked at the exit of the narrow segment of tissue (-90 degrees) while the counter-clockwise wave propagates around the circuit to terminate at the tissue narrowing. The third and fourth behaviors correspond to counter-clockwise reentry (CCWR) (Fig. 2C) and clockwise reentry (CWR) (Fig. 2D), in which a single pacemaker beat gives rise to a wave front continuously propagates in the counter-clockwise or clockwise direction, respectively. In these behaviors, tissue excitation is no longer driven by the spontaneous activity of the pacemaker cells, but rather is driven continuously by a circularly propagating wave front, provided that the time required to perform a full rotation around the circuit is less than the cycle length of the pacemaker cells.

**Fig 3:**
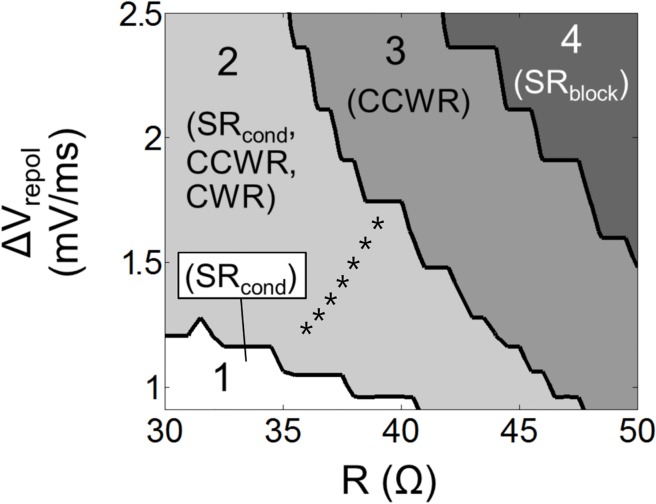
Regions of parameter space and their supported stable behaviors. The jagged edges reflect the discrete nature of the system as well as the relatively coarse sampling of parameter space. The asterisks correspond to the points in Table 1.

Each of the stable behaviors shown in Fig. 2 is a manifestation of the system operating at the bottom of a particular basin of attraction. These behaviors, however, do not all coexist for all sets of model parameters. For example, SR_cond_ (Fig. 2A) requires a wave front to propagate clockwise through the wedge, while SR_block_ (Fig. 2B) requires the wedge to block wave fronts traveling in either direction. Which of these stable behaviors is possible depends on the values of *ΔV*_*repol*_ and *R*. By running the model repeatedly from different initial states with *R* ∈ [30, 50] Ω and *ΔV*_*repol*_ ∈ [0.9, 2.5] mV/ms, each time noting which stable behavior the model eventually settled into, we found that there are 4 distinct regions in *R*-*ΔV*_*repol*_ space (Fig. 3). Region 1 supports SR_cond_ because the wedge supports propagation in both directions so that excitation beginning at the pacemaker site produces opposing waves that merely annihilate on the other side of the ring. On the other hand, neither CCWR nor CWR are possible because WL exceeds the path length of the circuit. Region 2 also supports SR_cond_, but now CWR and CCWR are possible as well because WL is less than the path length of the circuit so that circulating waves do not extinguish themselves on their refractory tails. In Region 3, WL is still less than the path length but now the wedge blocks waves traveling clockwise due to source-sink mismatch at the left-hand end of the isthmus. The latter condition prevents CWR, and unidirectional block implies that SR_cond_ is not supported because the clockwise traveling wave generated by the pacemaker cells will always terminate at the wedge, leaving the system in CCWR (reentry rate is always faster than the automatic rate so CCWR will supplant automaticity due to overdrive suppression). In Region 4, WL is again less than path length but now the wedge does not allow propagation in either direction. This makes reentry impossible so that SR_block_ is the only possible stable behavior.

Dynamic entrapment can arise in this system because three stable behaviors are possible in Region 2, while the adjoining regions (Regions 1 and 3) each support only one of these behaviors (Fig. 3). For example, if the system starts off in Region 1 (where it necessarily exhibits activity characterized by SR_cond_) but then encounters a perturbation that pushes it into Region 2, it will continue to exhibit SR_cond_ until the perturbation becomes great enough to push the system into Region 3 where it will be forced to assume CCWR behavior. If some ameliorating influence then causes the system to return to Region 2, CCWR will continue even though the very same system parameters were previously host to SR_cond_. In other words, this system exhibits hysteresis in its qualitative behavior.

**Fig 4:**
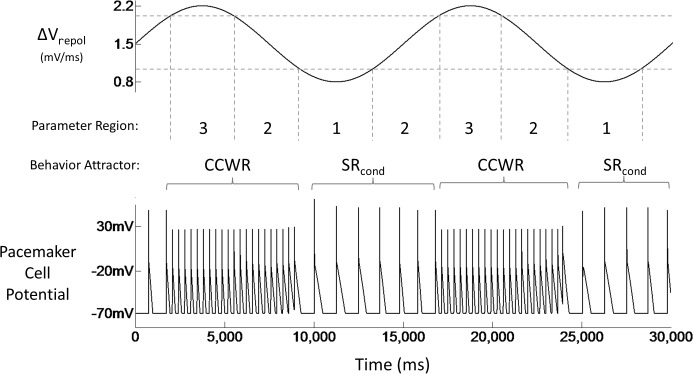
Mechanism of dynamic entrapment: parameter oscillation. A shift in behavior (SR_cond_ to CCWR) occurs when *ΔV*_*repol*_ rises above a critical value and the system enters region 3 of *R*-*ΔV*_*repol*_ space (at 2,000ms & 17,000ms); CCWR does not revert back to SR_cond_ despite reversal of *ΔV*_*repol*_ back into region 2 (at 5,000ms & 20,000ms). The inverse occurs when *ΔV*_*repol*_ falls below another critical value and the system enters region 1 of parameter space (at 9,000ms & 24,000ms); the system returns to SR_cond_ and remains there even when the system returns to region 2 (at 13,000ms & 28,000ms).

To explicitly demonstrate the hysteresis, we initialized the system in SR_cond_ with *ΔV*_*repol*_ = 1.5 mV/ms and *R* = 38 Ω, placing it in Region 2 of *R*-*ΔV*_*repol*_ space. We then varied *ΔV*_*repol*_ sinusoidally with an amplitude of 0.7 mV/ms such that the system alternately exited Region 2 into Regions 1 and 3 (Fig. 4). This gave rise to two critical transitions that can be understood with reference to Fig. 3. First, when the system exhibited SR_cond_ (the equivalent of normal sinus rhythm) and crossed from Region 2 to Region 3, its behavior immediately shifted to CCWR (the equivalent of, for example, atrial flutter). Upon returning to Region 2, the behavior continued in CCWR, an example of dynamic entrapment in a pathological attractor. The second critical transition occurred when the system continued from Region 2 into Region 1, where it necessarily reverted back to SR_cond_. This behavior persisted throughout the remainder of the *ΔV*_*repol*_ cycle during which the system returned to its starting point in Region 2. The period of oscillation was chosen arbitrarily because it is long enough to clearly show the hysteretic behavior but short enough so that individual R-R intervals are easily discernable. Longer oscillation periods, say, in the order of minutes could happen such as in response to exercise, and a corresponding plot would look essentially identical to Fig. 4 except on a different time scale and with the R-R intervals harder to resolve visually.

**Fig 5:**
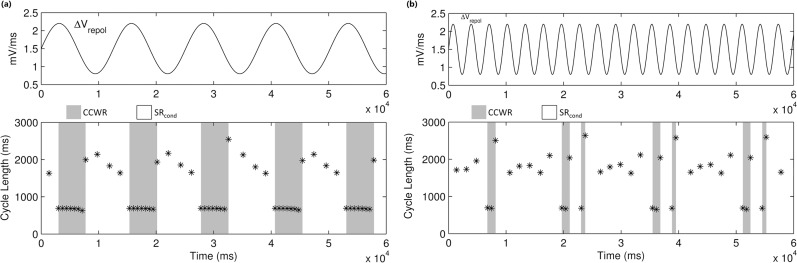
Effect of oscillation frequency on the occurrence of dynamic entrapment. The bottom panels show the cycle length of activation of a single cell in the circuit. Values below 1000ms correspond to rapid activation during CCWR. The cycle length during SR_cond_ varies with *ΔV*_*repol*_ as this alters action potential duration of the pacemaker cells. (a) The frequency of dynamic entrapment, i.e. transitions from SR_cond_ to CCWR (shaded in grey), corresponds to that of the oscillating parameter provided it is low enough. (b) Dynamic entrapment occurs irregularly and less frequently when the frequency is higher due to a beating effect, where the brief incursions of the parameter into Region 3 must occur while there is a wavefront attempting to traverse the isthmus from the right.

An even more complicated relationship between the electrogram and the underlying physiology is demonstrated by the model when the frequency of oscillation of the model parameter decreases to approach that of the reentry itself. This is exemplified in Fig. 5 which shows that, at sufficiently high frequencies, occurrences of dynamic entrapment become irregular as well as less frequent than the number of incursions into Region 3. Dynamic entrapment in this case requires that a wavefront attempt to traverse the narrow isthmus from the right while the parameter is in Region 3. At sufficiently slow frequencies, each incursion into Region 3 will contain at least one such event and lead to dynamic entrapment (Fig. 5(A)). When the frequency of parameter oscillation is sufficiently high, however, these incursions are very short and can occur while the system is between beats of SR_cond_. It therefore requires multiple incursions before one is appropriately timed such that unidirectional block occurs in the isthmus. The mechanism for converting CCWR to SR_cond_, however, has no phase dependence because a reentrant wavefront can collide with its refractory tail at any location in the circuit. The first incursion into Region 1 will therefore terminate reentry, resulting in short episodes of reentry separated by long intervals of sinus rhythm (Fig. 5(B)).

**Fig 6:**
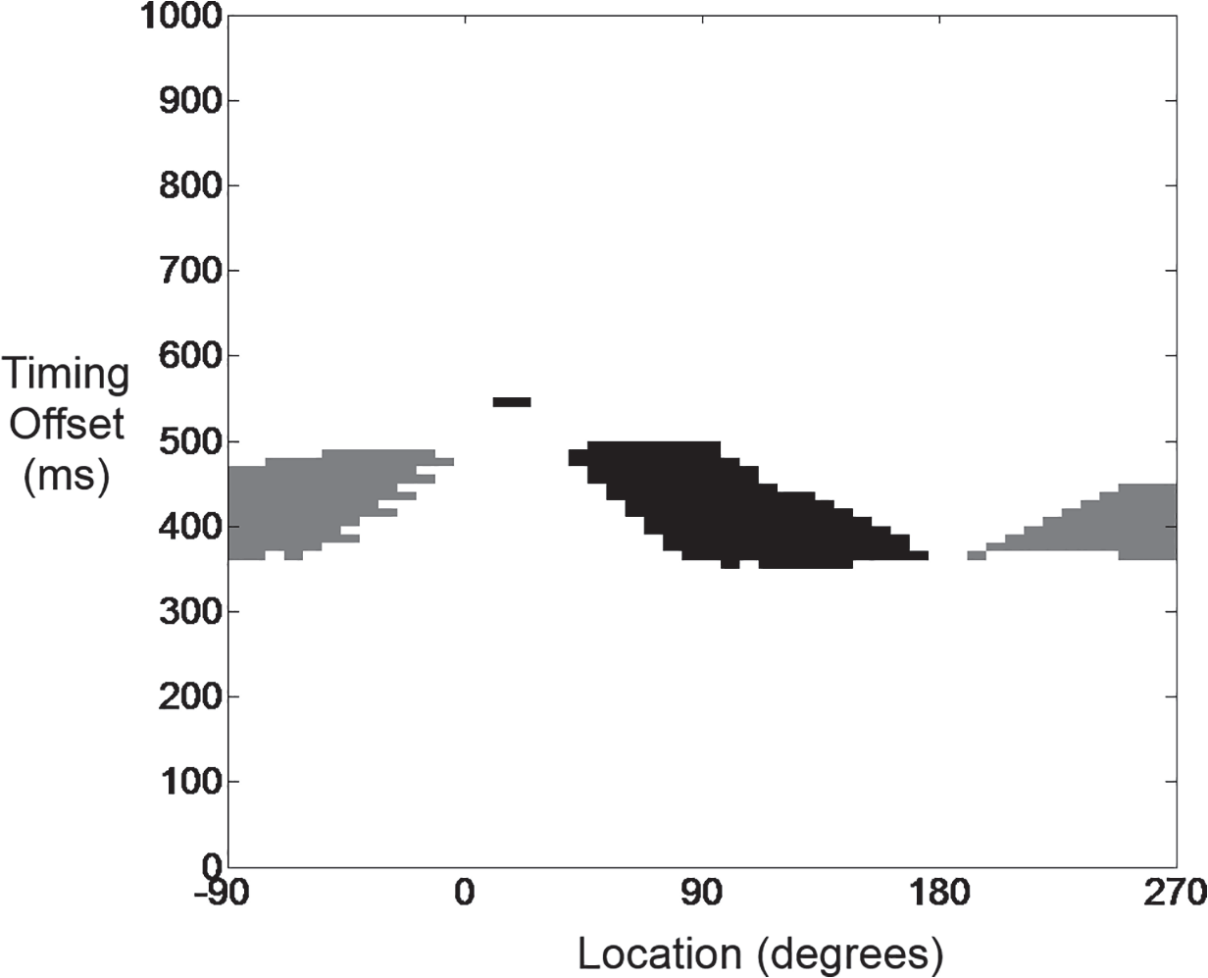
Inducibility of reentry by premature depolarization. Grey and black areas represent ectopic activations that successfully induced reentry (CCWR and CWR respectively), while white area represents those which failed to induce reentry and left the system in SR_cond_. Y-axis: coupling interval of external stimulus (ms) relative to pacemaker firing; x-axis: location of premature activation on disc (degrees).

We next investigated the ability of external perturbations, in the form of ectopic depolarizations, to push the system from sinus rhythm into reentry. Again, we initialized the system so that it exhibited SR_cond_ in Region 2, with *ΔV*_*repol*_ = 1.5 mV/ms and *R* = 38 Ω, and then determined the stable behaviors induced by ectopic activation at various locations around the circuit and timing relative to the pacemaker activation. Two distinct but contiguous patches corresponding to reentry emerged in the location-timing plane (Fig. 6), one patch corresponding to CCWR and the other to CWR. Ectopic activation in the remainder of the plane had no effect on SR_cond_ either because the cells at the ectopic site were refractory to re-excitation or because the two wave fronts that were spawned by the activation continued unimpeded around the circuit until they collided and annihilated each other. In both the reentrant patches, on the other hand, two waves were always spawned by the ectopic activation but one was blocked before it could travel far enough to encounter the other. Block occurred when one of the waves collided with the refractory tail of the preceding SR_cond_ wave, which happened in one of two ways. Sometimes the wave was spawned close enough to the wake the preceding SR_cond_ wave that its CV was increased by the elevated excitability of local cells that had not fully returned to resting potential, causing it to catch up to and terminate on the refractory tail of the SR_cond_ wave. Alternatively, if the waved chased the SR_cond_ wave into the wedge, where the latter could then slow down, again impaction on the refractory tail would occur.

The susceptibility of the system to dynamic entrapment caused by ectopic activation is proportional to the percent of the area, A_DE_%, of the location-timing plane that results in reentry (i.e. the gray and black areas as a fraction of the total area in Fig. 6). Table 1 lists A_DE_% for various combinations of *ΔV*_*repol*_ and *R* corresponding to locations in Region 2 beginning near the boundary of Region 1 and moving towards the boundary of Region 3 (Fig. 3), and demonstrates that susceptibility to dynamic entrapment increases as one moves away from the boundary with Region 1.

**Table 1:** Susceptibility of the system to dynamic entrapment caused by ectopic activation as a function of position in Region 2.

**R (Ω)**	36	36.5	37	37.5	38	38.5	39
**ΔV_repol_ (mV/ms)**	1.25	1.30	1.36	1.43	1.5	1.58	1.67
**A_DE_%**	0.2	3.9	5.04	5.54	6.3	6.58	6.64

## Discussion

The conventional linear view of physiology is that structure defines function, and conversely that function arises from structure. Accordingly, it seems natural to conclude that abnormal function, such as arises in disease, must somehow reflect a derangement of structure. Nevertheless, our understanding of nonlinear dynamic systems, a class to which living systems surely belong, makes it clear that the situation may not be quite so straightforward. Such systems are in principle capable of exhibiting multiple and quite disparate stable behaviors. This phenomenon is particularly well studied in reentrant neural networks [17], and these arguably serve as archetypes for biological networks in general [5]. Since innumerable biological networks are responsible for functions critical to life, this raises the possibility that some diseases might manifest when a biological network is stuck operating within a basin of attraction corresponding to abnormal function, a pathological mechanism called dynamic entrapment [3].

Perhaps the most intriguing aspect of dynamic entrapment is the hope of elucidation it offers for the pathophysiological basis of those many idiopathic diseases that currently evade mechanistic explanation. It remains to be seen, however, whether dynamic entrapment can occur in biological systems that are structurally perfectly normal; evolution may have already weeded out those with this capacity so that current living organisms are comprised of only the robust single-attractor variety [5]. Of course, the brain is in a special class in this regard because it clearly exhibits multiple stable states, and may even be susceptible to aberrant attractors such as have been proposed to explain epilepsy [18,19]. Our focus here, however, is on non-neural networks. There is little doubt that multiple dynamic attractors can exist in biological networks that have sustained some degree of structural damage. Indeed, the fact that uniform depolarization (i.e. direct current cardioversion) of a diseased heart can sometimes convert a pathological arrhythmia to stable sinus rhythm essentially proves this point. This then raises the key question as to what changes in structure can imbue the heart with the capacity to host multiple stable behaviors. Accordingly, our goal in the present study was to investigate how structural defects in the heart cell network might lead to dynamic entrapment.

To study this question we modeled the behavior of a ring of excitable tissue as has been done previously in numerous other studies [6–8,10,11]. We introduced a structural defect in the form of a local narrowing of the conducting tissue (Fig. 1). This mimics the well-known mechanism for unidirectional block due to the asymmetric electrical source-sink properties of a wedge-shaped isthmus between appropriately shaped segments of non-conducting scar tissue [20–22]. We showed that this system exhibits four distinct behaviors (Fig. 2), two of which cause each tissue cell to be excited only once following each pacemaker beat (i.e. mimicking sinus rhythm) while the remaining two involve perpetual re-excitation of each cell (correspond to reentry). The particular behavior the system exhibits depends on the intrinsic excitability of its constituent cells, which in the model is governed by the parameters *ΔV*_*repol*_ and *R*, as shown in Fig. 3. The crucial feature of the behavior landscape shown in Fig. 3 that leads to dynamic entrapment is Region 2, within which more than one stable behavior is supported. The system is thus capable of both normal and pathological behavior without any change in connectivity. Furthermore, since the adjoining regions (Regions 1 and 3) each support only one of these behaviors, if the system enters Region 2 from either Region 1 or Region 3 it will persist in whatever behavior it had upon entering. This creates hysteresis whereby the system behavior depends not only on the current excitability of the tissue but also on what that the excitability might have been previously.

Multistability is in fact a well-known phenomenon in cardiac modeling. It has been previously demonstrated in ring tissues [8,10,23] as well as in the break-up of spiral waves in heterogeneous media [24–26]. Hysteretic behavior has also been observed in the transition between meandering spirals and chaotic propagation when varying a model parameter [27]. Many of these prior studies employed sophisticated models of cardiac propagation. For our present purposes, however, we found it advantageous to employ a coarser-grained approach in order to reduce computation time and permit a broader exploration of parameter space. We ensured that our model retained the capacity to recapitulate essential phenomena of cardiac propagation, including multistability and hysteresis, on which dynamic entrapment critically depends. It is important here to highlight the difference between dynamic entrapment and the phenomena of multistability and hysteresis. The former refers specifically to the condition of a biological system having been pushed from a state of healthy function into an attractor corresponding to pathology, whereas the latter are requirements for this possibility.

For dynamic entrapment to be possible in a biological system capable of supporting multiple stable behaviors there has to be a mechanism for moving between different basins of attraction. Fig. 3 suggest how this might, in principle, occur. For example, suppose that the simple system we have considered here models the operating characteristics of an actual heart functioning within Region 2. The excitability of cardiac tissue, embodied in the model by the values of *R* and *ΔV*_*repol*_, is under the control of various factors including autonomic neural input and certain circulating hormones. It is thus conceivable that sufficient stress to an organism might produce transient shifts in the values of *R* and *ΔV*_*repol*_ that cause the system to make a brief excursion into Region 3 where it would adopt reentrant behavior that would then persist upon return to Region 2. Sinus rhythm is not restored in this case until a potentially much greater change in tissue parameters moves the system into Region 1. Alternatively, an appropriately timed ectopic depolarization can also convert sinus rhythm to reentry (Fig. 6). The latter mechanism, which has also been extensively studied by other investigators [6,8,28], correlates strongly with clinical observations that premature atrial complexes are a common trigger for initiating reentry in humans, e.g. [29,30]. The importance of heterogeneous tissue properties for the ability of ectopic activity to convert reentry to sinus rhythm has been highlighted by Sinha et al. [31,32]. However, heterogeneity in these studies was created by changing electrical properties rather than the tissue’s structure as we do here.

Our results also suggest that the capacity to suffer from dynamic entrapment is a matter of degree. Specifically, when our model is caught exhibiting reentrant behavior in Region 2, the farther away it is from the border of Region 1 (and thus presumably the more diseased it is) the greater are the changes in tissue parameters needed for it to revert to sinus rhythm (Table 1). In other words, a mildly diseased network would be expected to be less prone to dynamic entrapment, or to more easily escape from the clutches of a pathological attractor, than would a severely diseased network. This can lead to some interesting consequences for the appearance of symptoms because parameters such as *R* and *ΔV*_*repol*_ invariably exhibit some degree of variation with time. This might cause the system to oscillate randomly between the regions shown in Fig. 3, giving rise to arrhythmias that are intermittent and not easily related to changes in system parameters, as illustrated in Fig. 4. Even periodic oscillations can lead to irregular alternations between healthy and pathologic behaviors when the mechanism of entrapment is phase dependent, as illustrated in Fig. 5. The hysteretic behavior in our model thus predicts a highly inconsistent and potentially confusing relationship between clinical symptoms and underlying pathophysiology. Furthermore, sustained tachycardia triggers remodeling of cardiac tissue that, among other things, shortens wavelength and decreases conduction velocity [33–35]. Such remodeling could cause the mean position of the system in the *R*-*ΔV*_*repol*_ plane to migrate away from the boundary with Region 1, causing the apparently random appearances of aberrant rhythms to become more frequent as disease progresses. This scenario is, in fact, reminiscent of the natural history of atrial fibrillation, which frequently begins in an intermittent “paroxysmal” form and then typically progresses to become persistent and eventually permanent [36,37].

It must be pointed out that we are not the first to model pathogenesis as a manifestation of nonlinear system dynamics. A notable prior example of this concept is that of *dynamical disease*, with particular reference to leukemia, developed by Mackey and coworkers [38,39]. Here, however, healthy or pathologic behavior is purely a function of current parameter values, so the hysteresis necessary for dynamic entrapment is absent. Interestingly, we observe a similar phenomenon in our model system when it moves through *R*-*ΔV*_*repol*_ space between Region 3 and Region 4 (Fig. 3), where behavior is entirely determined by current location.

A disease that manifests as dynamic entrapment poses particular challenges in terms of therapy, for which there appear to be two options. One is to alter the system (either its structure or the values of its parameters) in such a way as to eliminate aberrant basins of attraction. As an example, anti-arrhythmic drugs may prolong *WL* [40,41], which in our model could have the effect of pushing the system into Region 1 where it has no choice but to behave normally. Another example is the creation of strategically placed non-conducting scar tissue (via catheter ablation) to change the physical structure of the system. In our model this could be applied to eliminate conduction through the wedge (Fig. 2), completely and permanently altering the topology of the *ΔV*_*repol*_-*R* landscape (Fig. 3) to produce a system in which only sinus rhythm is possible. The other therapeutic approach to dynamic entrapment is to persistently push the system back into the healthy attractor whenever it gets stuck elsewhere. As an example, defibrillators or anti-tachycardia pacing convert reentry to sinus rhythm, which would be efficacious in our model if it were located in Region 2 where such conversion could have sustained benefits.

In conclusion, we have used a numerical model to demonstrate how dynamic entrapment can feature in the pathogenesis of arrhythmias as a result of structural defects in the network of excitable cells that comprises cardiac tissue. This raises the possibility that dynamic entrapment might also underlie other complex diseases such as those involving chronic inflammation or auto-immunity, which could have major implications for therapy. The evidence thus far suggests that dynamic entrapment only occurs in biological networks or other nonlinear biological systems that have already sustained some kind of structural damage, and that completely normal systems are robust against this type of affliction. It remains to be seen if there are some diseases manifesting dynamic entrapment in biological networks that are completely normal. In such cases there would be no evidence for the existence of an instigating factor, which would make causation opaque and prevention extremely challenging.
